# The Midwives Bill

**Published:** 1902-07-12

**Authors:** 


### The Hospital

A Journal of

The Medical Sciences and Hospital Administration.

Vol. XXXII.—No. 824. Edited by Sir Henry Burdett, K.C.B., and Solomon C. Smith, M.D., M.R.C.P. [July 12, 1902.

### The Midwives Bill.

The important amendment to the Midwives Bill which was carried by Dr. Ambrose on the second reading in the House of Commons, and by which the habitual practice of midwifery for gain by unregistered women will become a penal offence after January 1st, 1910, has since been accepted in Committee by the House of Lords, where the Bill now awaits only the third reading. We may, therefore, look forward to the near approach of a time when women who, in the judgment of the Board to be created by the Act, are sufficiently qualified for the ordinary duties of attendance upon natural labour will be available in every district of England for all who require their services; and we may also look forward to a future, near in the history of a nation, if somewhat remote in that of an individual, when the unregistered midwife will be an offender against the law, and will be rendered liable to its penalties. It is perhaps not too much to say that the interests of the poor are always more carefully considered and more effectually safeguarded in the Upper than in the Lower House, and in the debate on Friday last more than one noble lord expressed anxiety lest the Bill as a whole, or Dr. Ambrose's clause in particular, should inflict hardship upon poor women by hindering them from having recourse to the friendly help of neighbours. Any danger of this kind seems, however, to be effectually provided against by the wording of the clause, which refers only to "habitual attendance for gain," and which would, of course, be interpreted strictly by any magistrates who were called upon to apply it. The real argument in favour, not only of the Bill, but also of the penal clause, was furnished by Lord Spencer, who pointed out that the general mortality of women in childbirth amounts to 1 in 200 in England and Wales, while the mortality in lying-in hospitals is only 1 in 600; so that a reduction of the general to the hospital rate would effect a saving of about 3,000 maternal lives every year. Since the introduction of antiseptic precautions into hospitals these institutions offer, of course, many advantages over the homes of the very poor, and it is too much to hope that the latter will all at once be rendered salubrious, or that those dwelling in them will be preserved from the numerous risks incidental to their poverty; but still there is much reason to regard the present mortality as being very largely due to gross neglect or to inexcusable delay, and against both of these conditions the new midwives should be able to afford a considerable increase of security.

It is an interesting question for the medical profession, and by no means an unimportant one as regards the public, how far the new midwives are likely to supersede qualified practitioners, either male or female, in the lying-in rooms of patients who are able to pay reasonable fees for the benefit of distinctively "medical" attendance. No doubt there will be some effect of this kind; but we apprehend that it will be very small in amount, and wholly unimportant as regards any general bearing upon the demand of the public for professional services. The midwives are likely, on the other hand, to relieve the doctor, and especially the busy country doctor, of a considerable amount of fatiguing and ill-remunerated work. He will feel justified in leaving a nurse, who is also a registered midwife, in charge of protracted cases which would otherwise detain him for long periods of time; so that he will obtain, in fact, the services of what might perhaps be called a "semi-skilled" assistant, capable of a good deal of management under his supervision, and capable, also, of keeping him informed of the real progress of events. On the whole, the gain to the doctor will probably, as a rule, preponderate over the loss; but it is none the less desirable for members of the medical profession to observe that their interests have not been regarded, in either House of Parliament, as matters calling for legislative protection, or, indeed, even for mention in the course of the debates. The questions at issue have been discussed upon public grounds, and upon public grounds alone. Such pleas and arguments as those which have been put forward by the leading agitators in what may be described as the "trades union" section of the medical profession have been treated in Parliament with the silence of contempt. Possibly, indeed, the profession as a whole is scarcely better acquainted with public opinion than these pinchbeck "leaders." Every doctor is personally popular in his own *clientèle*, and, when he denounces midwives and quacks and quack medicines, everybody is civil to him, and bows politely to his superior knowledge. As soon as his back is turned, they send round to the neighbouring chemist for the quack medicine, or they drive over to the market town and obtain an interview with the quack himself. As for the doctor's anathemas, they are only "pretty Fanny's way."

The truth is that the medical profession—in bygone times more, perhaps, than at present—has cultivated mystery; and the public have come to regard mystery as being largely synonymous with humbug. We have neglected to educate our employers, and so we are liable to be treated by them with a contumely which is the direct offspring of ignorance.

